# Electronic knowledge books (eK-Books) as a medium to capitalise on and transfer scientific, engineering, operational, technological and craft knowledge

**DOI:** 10.1371/journal.pone.0299150

**Published:** 2024-05-17

**Authors:** Cédric Baudrit, Christophe Fernandez, Julien Couteaux, Amadou Ndiaye

**Affiliations:** 1 Institut national de recherche pour l’agriculture, l’alimentation et l’environnement, Institut de mécanique et d’ingénierie, Talence, France; 2 Université de Bordeaux, Institut de mécanique et d’ingénierie, Talence, France; West Pomeranian University of Technology, POLAND

## Abstract

The capitalisation on and transfer of technological, engineering and scientific knowledge associated with empirical know-how is an important issue for the sustainability and development of manufacturing. Indeed, certain sectors of industry are facing the increasing ageing of the labour force, recruitment difficulties and high staff turnover, leading to a loss of knowledge and know-how. In a context of numerical and digital transition and the migration of processes to industry 4.0, one of major challenges manufacturers face today is their capacity to build intelligent platforms for acquiring, storing and transferring their know-how and knowledge. It is crucial to create new media and tools for staff training and development capable of capturing knowledge and reusing it to create a project history through expertise and data collection. This paper presents the methodology and guidelines for implementing electronic knowledge books (eK-Books), along with their uses. The eK-Book is a semantic web-based hypertext medium (channel) allowing stakeholders to capitalise on, structure and transfer knowledge by using concept maps, process maps, influence graphs, downloadable documents, web pages and hypermedia knowledge sheets. They are intended for engineers, expert or novice technicians, manufacturers, sector coordinators and plant managers, as well as trainers and learners. They are usable and manageable in all types of environments and with different levels of accessibility. This paper highlights (1) the transfer knowledge capacity of eK-Books and (2) their usability in two agri-food sectors namely (1) the cheese sector with protected designation of origin (PDO) and protected geographical indication (PGI), and (2) the butchery and cold meat sectors.

## Introduction

Faced with the increasing ageing of the labour force in industry, the migration of processes to industry 4.0 and recruitment difficulties in certain sectors, one of major challenges for manufacturers today is their capacity to build intelligent platforms for acquiring, storing and transferring their know-how and knowledge. Increasing automation and decision-making guided by data may also lead to a reduction in human labour in the production process, which may contribute to the disappearance of jobs, the reduction in expertise and the loss of know-how in manufacturing organisations. However full automation seems unlikely as machines cannot completely replace humans, who will continue to play a key role in industries [1]. For instance in France, the agri-food sector is struggling to recruit (e.g., more than 3000 permanent positions are left vacant in the dairy sector each year), leading to a loss of knowledge and know-how, and high staff turnover. Weakened by this context, industry must also deal with increasingly restrictive standards, while having to rely on a growing quantity of generic knowledge and massive data, due to scientific and technological advances. The capitalisation on and transfer of technological and scientific knowledge and empirical know-how is an important issue for the sustainability and development of manufacturing. Industrial sectors must acquire digital tools to structure their knowledge domain and then develop and exploit these knowledge bases. Processes are based on a multitude of skills, know-how and experience forged over time. Through their knowledge and know-how, manufacturers adapt their practices according to variations in the properties of raw materials and the characteristics of final products. This positioning generates a wealth of know-how among stakeholders based on their own experience, which is essentially passed on through on-the-job training. Moreover, the ways in which knowledge is transmitted vary from one individual to another. Organizing the transmission of knowledge and know-how within the company first and foremost means giving employees a methodology for transferring expertise in an efficient and sustainable manner.

Knowledge comes in two formats: declarative and procedural. The first refers to knowledge, to what we know; and the second to know-how, to skills that we are able to perform [2]. Declarative knowledge is explicit and static whereas procedural knowledge is dynamic and may be either explicit or tacit [3]. One of the main challenges of the manufacturing industry is to be able to transform individual tacit knowledge into explicit collective knowledge for the entire workforce. From a bibliometric analysis, Anand et al., [4] conclude that by conducting research into capitalisation, the storage and retrieval of explicit and tacit knowledge could be beneficial to small and medium-sized enterprises (SMEs) in the long term. The main challenge is to create new media and tools for staff training and development by capturing knowledge and reusing it to create a project history through the collection of expertise and data [5]. Without knowledge transfer tools, manufacturing practices are passed on from experts to novices through observation and imitation. Experts acquire expertise stemming from years of experience and are often incapable of explaining the scientific rules or all the relevant phenomena involved in their expertise. The need to share knowledge between universities and industry has gradually become clear in recent years [6, 7].

This paper presents the use of electronic knowledge books (eK-Books) based on a semantic network, for collecting, structuring and remobilising the knowledge and know-how of any sector or company in a context of numerical and digital transition. The article proposes an easily usable integrated electronic platform designed for the heterogeneous and complex sharing and transfer of knowledge. A generic and formal unified framework is defined allowing users to handle knowledge stemming from different sources or domains and expressed in different forms, different formats and at different scales. The paradigm of eK-Books provides the architecture of digital teaching based on the results of research [8]. The proposed structure of eK-Books in this article is very similar to the human cognitive architecture. They revolutionize the way people read and access information, offering a versatile and accessible alternative to traditional printed books. Through various hyperlinks, they offer a proactive, dynamic, non-linear reading experience that enables learners or users to access specific information or construct their own reading path in a flexible manner based on their interests or needs. They facilitate adaptive learning allowing industrial firms to tailor training content to individual learning styles, enhancing the effectiveness of knowledge transfer methods. The proposed semantic associated with eK-Books allows industrial firms to break down knowledge silos to adopt a more holistic approach to knowledge capitalization and transfer. The paradigm of eK-Books allows breaking down barriers to education and opportunities, promoting social equity. The methodology established for designing eK-Books in this article facilitates incremental collaborative updates and upgrades, both in their structure and content. It is accessible to both experts in knowledge engineering and those whose knowledge in knowledge representation or computer science is limited. Two eK-Books were designed and tested in the agri-food sector: (1) the cheese sector with protected designation of origin (PDO) and protected geographical indication (PGI), and (2) the butchery and cold meat sector.

The aim is to:

- Avoid the loss of knowledge and know-how;
- Formalise good practices and capitalise on reusable knowledge to make transfers durable;
- Encourage the sharing of expertise on a daily basis;
- Optimise and ensure the reliability of handovers between people
- Ensure the sustainability of knowledge and skills;

The tool has the following advantages:

- Usable by all stakeholders in all types of environments and with different levels of accessibility;
- Minimisation of knowledge capture errors;
- Ergonomic interface to acquire and transfer knowledge;
- Integrated and manageable tool in the company;
- Computer assisted construction and updating of the Knowledge Book.

The article is organized as follows. The following section reviews related works; the electronic knowledge book section details the materials used in the eK-Book models; the electronic knowledge book design process section proposes a methodology to design eK-Books and the last section highlights the capacity of eK-Books to transfer knowledge in agri-food sectors.

## Related works

In this paper, knowledge corresponds to general concepts representing a set of things with a common meaning in a specific field and data corresponding to specific instances [9].

The loss of operational, craft and tacit knowledge is now always mitigated by recording this knowledge via internal reports, succession plans and storytelling, passing it on during on-site training, or formalising it by means of rules and model systems [10, 11]. These approaches remain informal and rely on workers’ communication skills and the availability of seniors and experts, and having inherent updating difficulties, for instance in rule-based systems.

Numerous studies on knowledge transfer and knowledge management have been carried out in both the private and the public sector, in order to discover the best ways to transfer knowledge according to multiple factors [12, 13]. One of the best-known knowledge management methods for acquiring expert knowledge from an enterprise or organisation is the MASK methodology (Method for Analysing and Structuring Knowledge). It leads to the creation of a “knowledge book”, which is a structured synthesis of knowledge essentially composed of knowledge diagrams, as well as publications, references, and all kinds of documentation [14]. This method focuses on business processes rather than industrial transformation processes. The resulting diagrams may prove difficult for certain stakeholders, for example craftspeople, to understand and take ownership of [15]. Moreover, MASK is not suited to scientific knowledge that includes simulating and reasoning models and results published in specialist reviews. Suciu et al., [16] propose a nonlinear network of concept maps linked via ontological relations to represent and transfer scientific knowledge to industrial firms. The REX (Return of EXperience) method [17] is an experience feedback-oriented method which assumes that lessons are learned from events and failures occurring during activities. REX is often focused on the technical factor, thereby underestimating the human factor, and neglects the aspects of an organisation’s intellectual capital. CommonKADS [18] aims to develop searchable and questionable expert systems which will represent the cognitive process of experts. However, this methodology requires a specific programming language which is unusable by non-experts, involving the manipulation of a mix of notations adopted from the Unified Modelling Language (UML), Structured Analysis and Design Technique (SADT), along with flow charts symbols.

Knowledge graphs [19] have recently garnered significant attention from both academia and industry, and seems undoubtedly to be one of the best approaches for capturing tacit and explicit knowledge. There is still no formal definition of the knowledge graph [20]; it relies on the formalism of a conceptual graph [21]. A knowledge graph may be viewed as a graph database, that is, semantic knowledge called a knowledge base where relationships between facts are formally described by the ontology [22]. However, knowledge graphs remain difficult for non-specialists to understand because they require specific programming languages based on specific formats such as RDFS (Resource Description Framework Schema) [23] and its evolution, OWL (Web Ontology Language) [24, 25] which is mainly applied in connection with the Semantic Web framework [26]. A more accessible and practical formalism for non-specialists is the concept maps [27], which offers a more natural and visual way to describe a domain in diagram form using relationships between concepts. It is more intended to support knowledge transfer and learner learning [28–30]. Lin et al., [31] propose, for instance, a concept map-based framework to help business, especially heavy industry, to retain domain-specific tribal knowledge.

## Electronic knowledge book (eK-Book)

**Fig 1:**
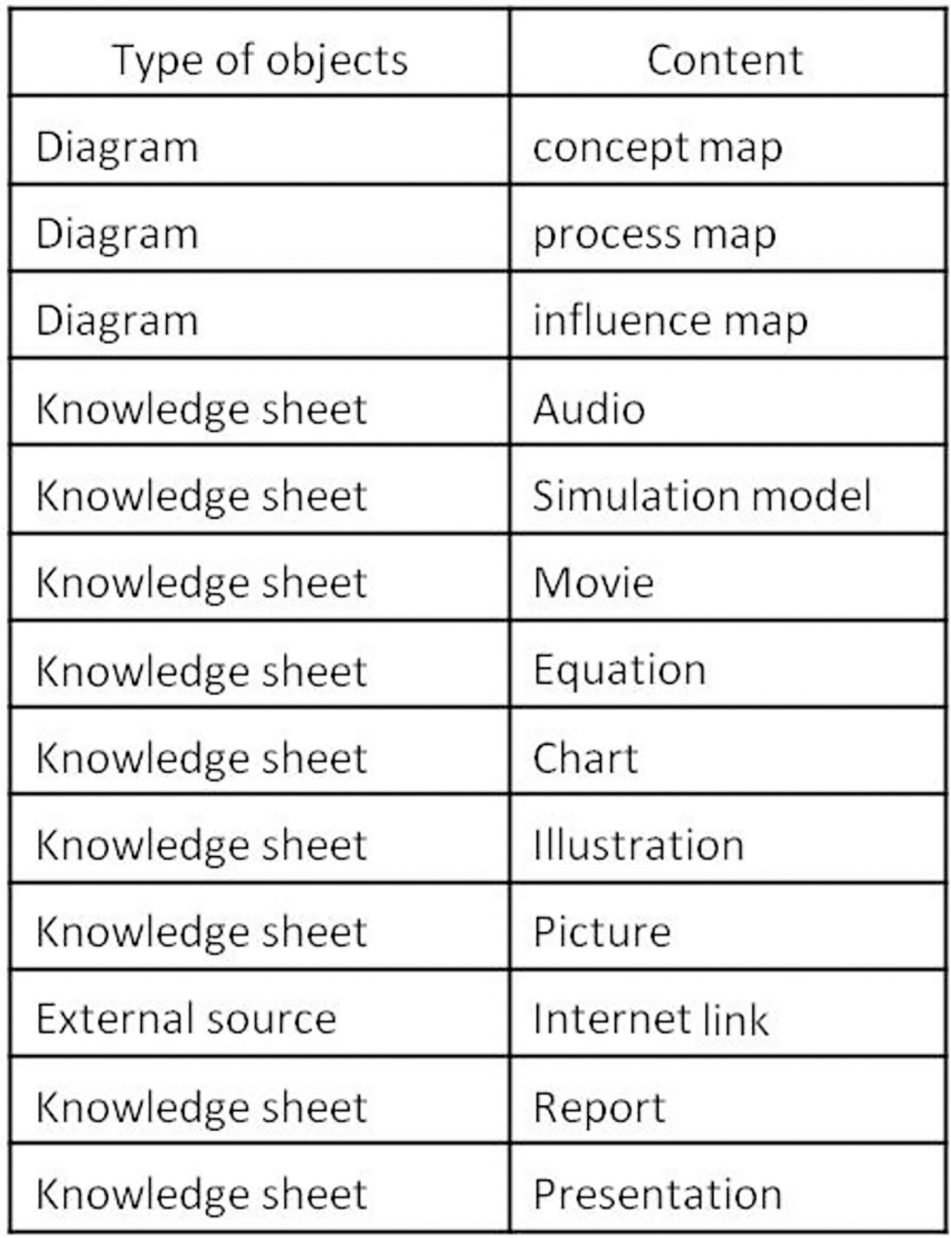
Objects used in an eK-Book associated with their content.

The electronic knowledge book (eK-Book) is a semantic web-based hypertext medium (channel) allowing stakeholders to structure, capitalise on and transfer knowledge by using concept maps, process maps, influence graphs, hypermedia knowledge sheets, web pages and downloadable documents. Concept maps (respectively process maps, knowledge sheets) allow declarative knowledge (respectively procedural, explicit and tacit knowledge) to be represented or, for tacit knowledge, to be captured. Concept maps, process maps, influence graphs, knowledge sheets, web pages and downloadable documents are interconnected by hyperlinks. Fig 1 displays the types of manipulated objects in an eK-Book associated with their contents. An eK-Book relies on a standardised domain vocabulary which is available in a glossary.

### Hierarchical structure of concepts–canonical cmaps

**Fig 2:**
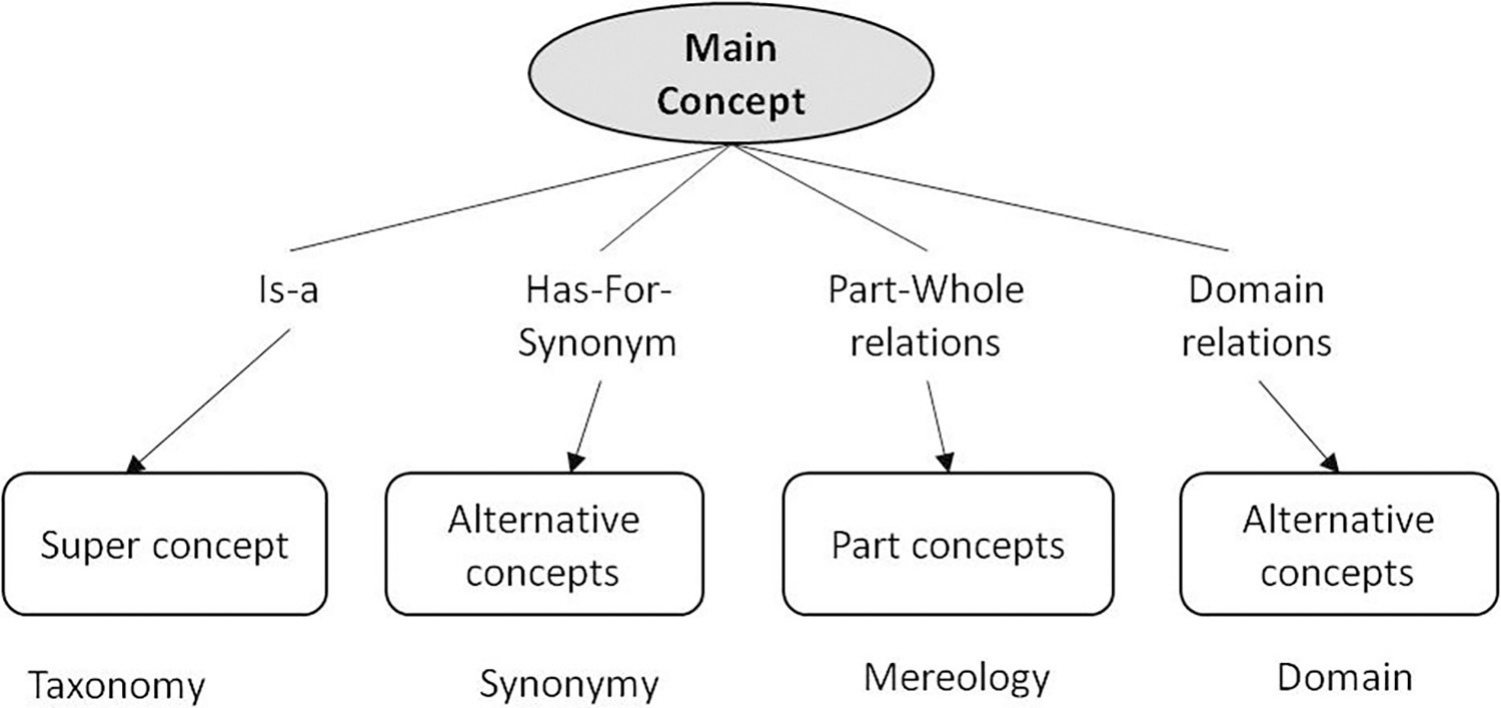
Generic canonical concept map.

Concept maps (Cmaps) with a hierarchical structure on 2 levels are chosen to allow users to acquire a large quantity of knowledge without too much disorientation or an overly large cognitive load when they read them [29]. In a concept map, nodes represent concepts that are connected with arcs expressing the relationships that exist between them. Cmaps form the skeleton of the eK-Book within a semantic network; they are each composed of a main concept described by other concepts via ontological relations. Each of these concepts can benefit from its own cmap as the main concept if necessary. A canonical concept map has been defined as a hierarchical diagram that allows users to describe each main concept according to four types of semantic relations, namely taxonomy, synonymy, mereology and domain relation (see the generic canonical cmap in Fig 2). The progressive description of each main concept allows users to stop navigating at a level of detail sufficient for their understanding. Thanks to this representation of knowledge, from general to specific, the user can browse the book by means of hyperlinks until the desired detail level is reached.

- **Taxonomy** is an ontological relation connecting a sub-concept to a concept via the ***Is-a*** relation; it is the primary ordering relation for class hierarchies. Specialisation/generalisation is the most intuitive method to organise the knowledge hierarchically. Such a hierarchy is useful for presenting limited views of the model that are easily interpretable by humans [32]. In this work, the ***Is-a*** relation is a generalisation relation allowing the concepts of a domain to be organised from the specific to the general [33]. This relation has a central role in knowledge representation, ontology engineering, and knowledge organisation, most notably in the construction and the primary ordering relation for vocabulary hierarchies. The inverse relation for ***Is-a*** can be defined trivially as follows: A ***Has-subclass*** B.
- The synonymic relation ***Has-For-Synonym*** makes it possible to specify equivalent alternative concepts in a given knowledge domain. The synonymy relation allows eK-Book designers to use the name of the concept corresponding to the professional vocabulary, allowing for a more efficient transfer to the user’s community.
- **Mereology** is the theory of the part–whole relation allowing an entity to be link to its parts [34]. Based on the works of [35–38] on the development of a formal taxonomy of part–whole relations, the different types of relations selected for this work are displayed in the Table of S1 File. Three illustrations of the relevance of these relations and their usability in knowledge capitalisation and transfer are given below:

**“**Cheese making ***Involves*** {milk preparation, adding coagulant, draining, salting, ripening, conditioning}”relates the phases (parts) to their encompassing process, “cheese making” (whole). Such a relation provides pertinent information that allows new learners in dairy to learn the appropriate steps to make cheese.

The first step in making cheese is acidification. During this stage, rennet is added to milk, which changes the lactose (milk sugar) into lactic acid leading to the beginning of coagulation. An acidification fault may be observed due to the presence of inhibitors such as antibiotics in the milk. “Milk ***Contains*** antibiotic” relates the antibiotic (a part) to the milk, but the antibiotic shares no parts in common with the milk. This relation may be useful to operators as it may facilitate the diagnosis of the slow acidification phenomenon, for instance.

**“**Spillway **Is-a*-Functional-Component-of*** dam” relates the “dam” system to one of its components, the “spillway”. This relation may be useful to engineers, allowing them to carry out and check the design of the spillway because when undersized, for instance, it may cause a dam failure due to its inefficiency in evacuating water.

- **Domain semantic relations** are specific relationships to the knowledge domain represented. This category of relations includes causal, temporal, procedural relations, etc. Some domain relations have come to be standardized, such as the Open Biomedical Ontologies [39] that proposes a collection of relations intended for use across life science domains. Verbs are generally used to express clearly and unambiguously the relationships between concepts. A set of semantic relations has been extracted from existing ontologies (see Table in S2 File) and adapted (1) for the domain of industrial transformation processes from raw materials to final products and (2) to have a common meaning for all stakeholders (experts, scientists, operators, etc.).

### Process map

**Fig 3:**
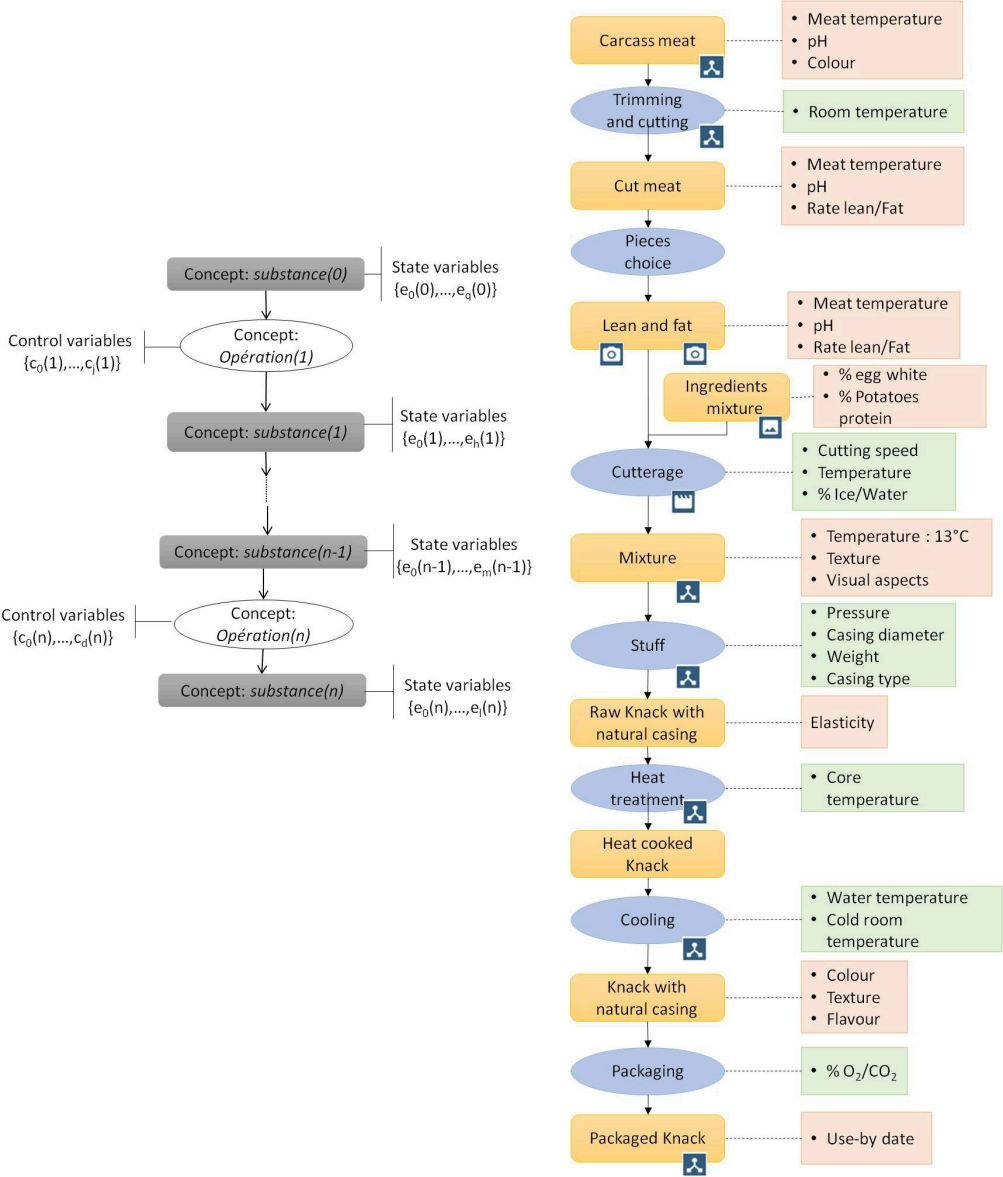
Representation of a generic multi-step process on the left and a simplified extraction of the sausage Knack sausage-making process on the right.

Ndiaye et al., [40] developed a formalism to represent a multi-step process (see Fig 3). The steps are ordered by unit operations and the number *n* of steps is finite. Each step *i* (*i* ranging from 1 to *n*) corresponds to a unit operation characterised by a set of input state variables **e**_***i*-1** =_ {e_*i*-1,1_, …, *e*_*i*-1,*u*_} and a set of output state variables **e**_***i***_ = {e_*i*,1_, …, e_*i*,*v*_}. Each unit operation is controlled by a set of control variables **c**_***i***_ = {*c*_*i*,1_, …, c_*i*,*m*_} meaning that output variables **e**_***i***_ depend on ***e***_**i-1**_ and ***c***_***i***_.

### Knowledge sheet

**Fig 4:**
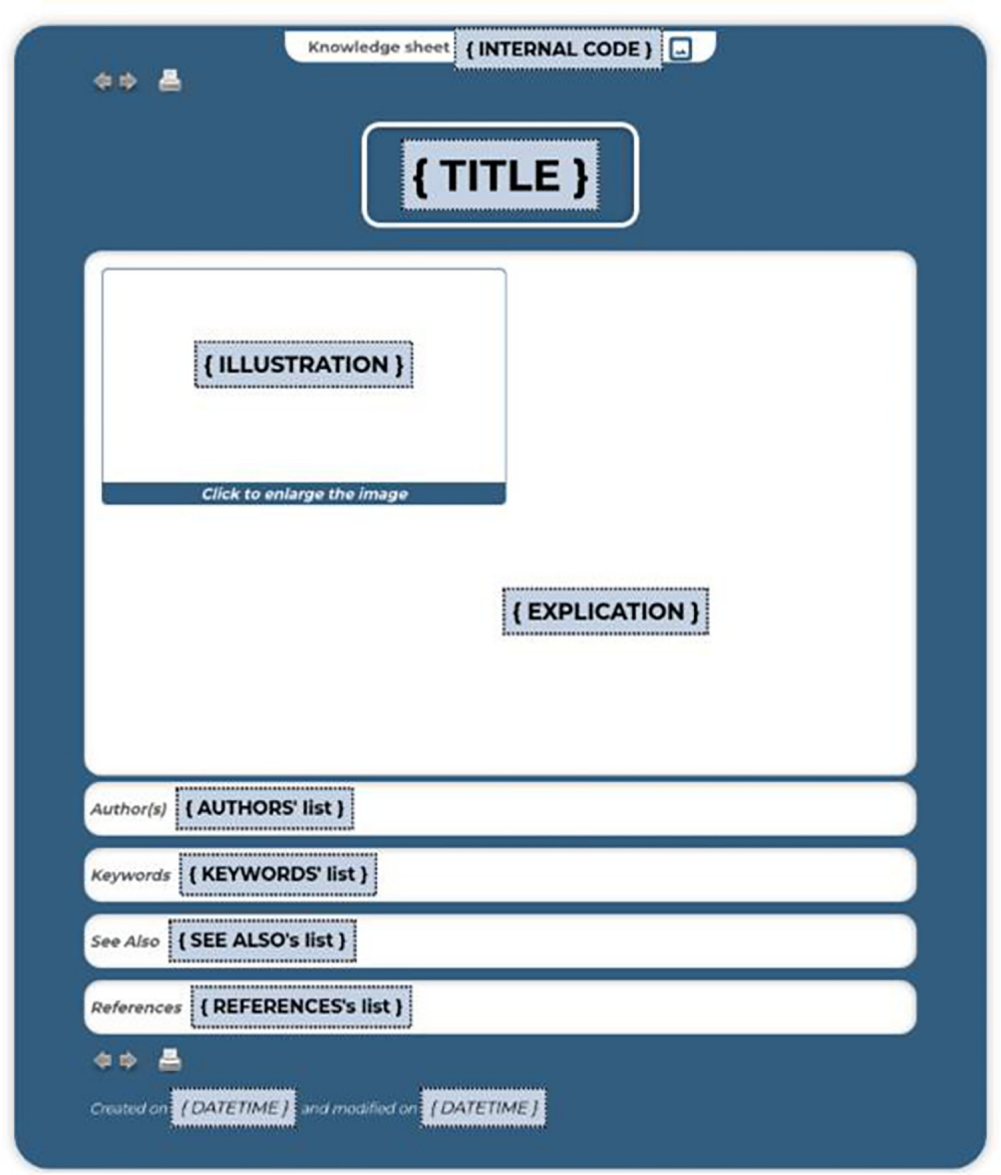
Illustration of the generic knowledge sheet template.

**Fig 5:**
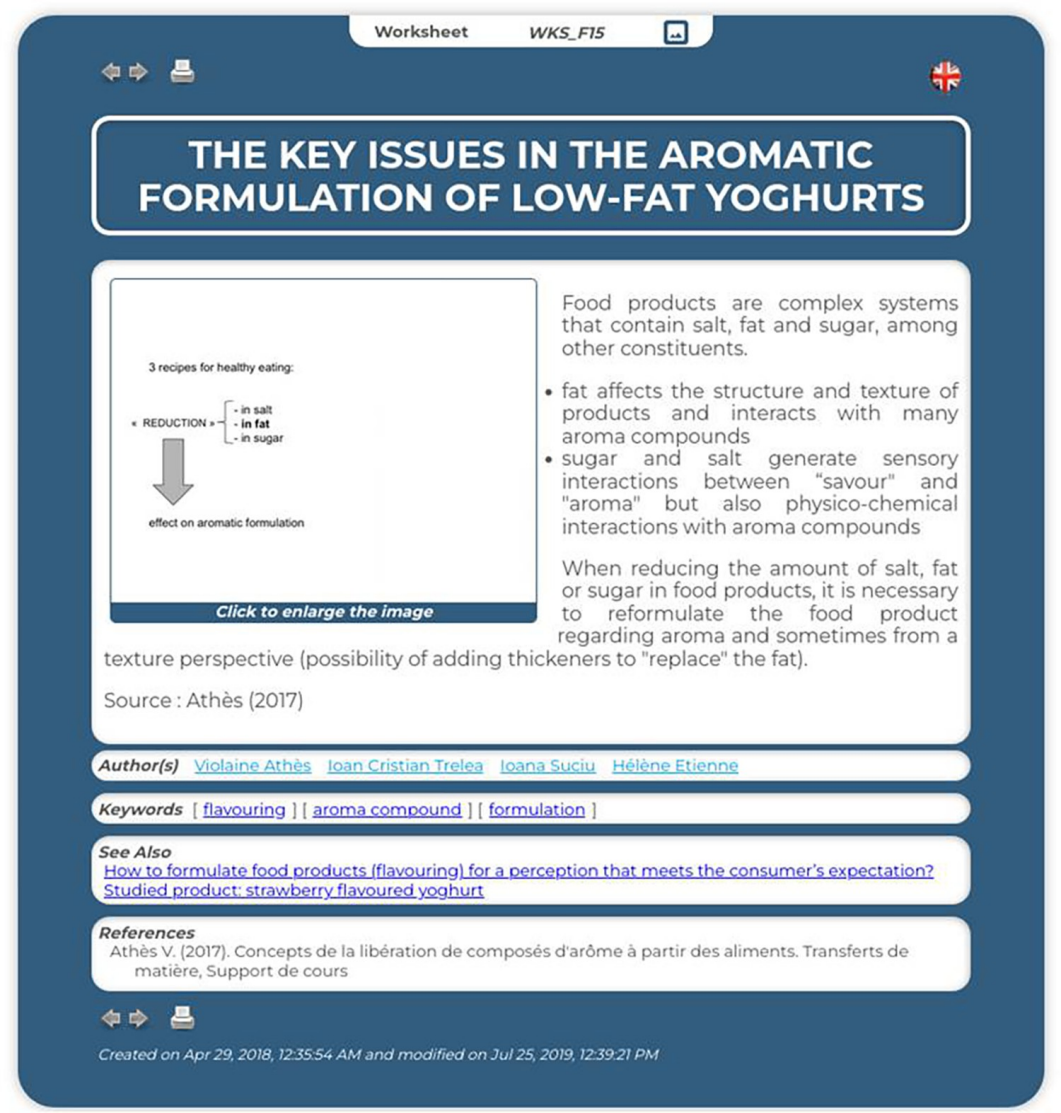
Illustration of an instantiated knowledge sheet about the key issues in the aromatic formulation of low-fat yoghurts.

The knowledge sheets are composed of a predefined set of clickable descriptive fields: *title*, *illustration*, *explanations*, *date of creation*, *authors*, *keywords*, *see also* and *bibliographic references* (see generic template in Fig 4 and a section of an instantiation in Fig 5). The illustration can be a video, a sound, a photo, a drawing, a graph, a table, an equation, a simulation tool, a decision support system, *etc*., and is a link to a document that can be consulted online.

The explanation is a text that can be formatted (font, bold, italic, colour, etc.); links to external sites or other applications can also be added into the body of the text. All information about authors (email address, web page, speciality, etc.) may be accessible via hyperlinks. Each keyword contained in the glossary can be clicked on to obtain a contextualised definition of the word. The sheet also collects links to semantically related records that provide additional information. The bibliographic references display those cited in the explanation and others that provide additional information. The bibliographic references display the ones cited in the explanation and others that provide additional information. They also can be clickable to access a web page or to open a linked document.

### Influence graph

**Fig 6:**
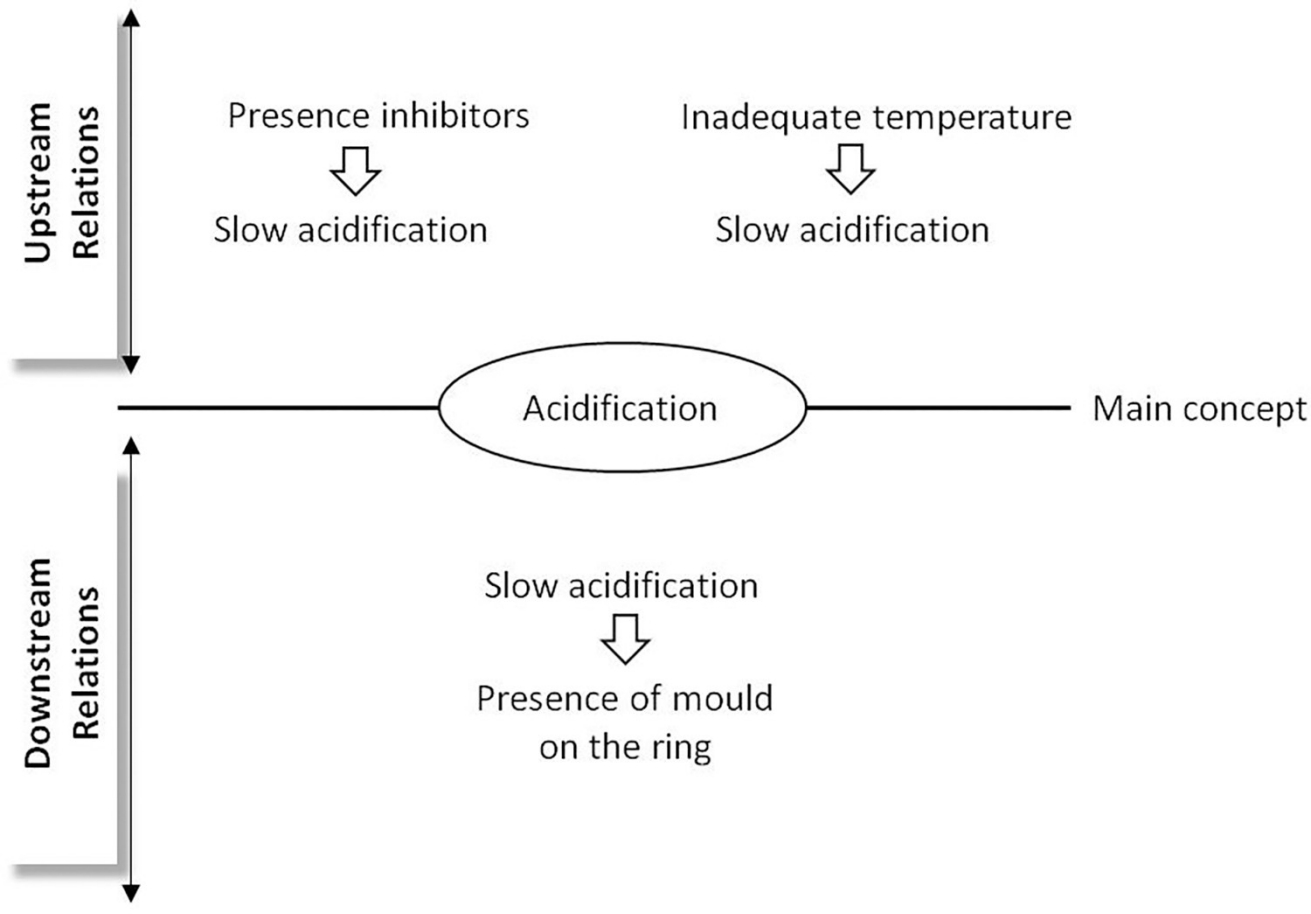
Influence graph of acidification during cheese making.

Influence graphs in eK-Books propose a vertical representation of influences between concepts, with upstream causal concepts influencing a central main concept which consequently influences downstream concepts (see example in Fig 6). An influence graph is relevant to represent the variables that influence a unit operation and the variables that it influences in order to facilitate the analysis, transformation and valorisation of products. In an analysis, transformation or valorisation process, a unit operation allows the product to be transformed from one state (operation input) to another state (operation output) in relation to the settings made during the operation via specific variables known as control variables. The result of the operation is described in terms of a set of state variables. In this case, the influence graphs make it possible to represent the influence of the control variables on the state variables.

## Electronic knowledge book design process

**Fig 7:**
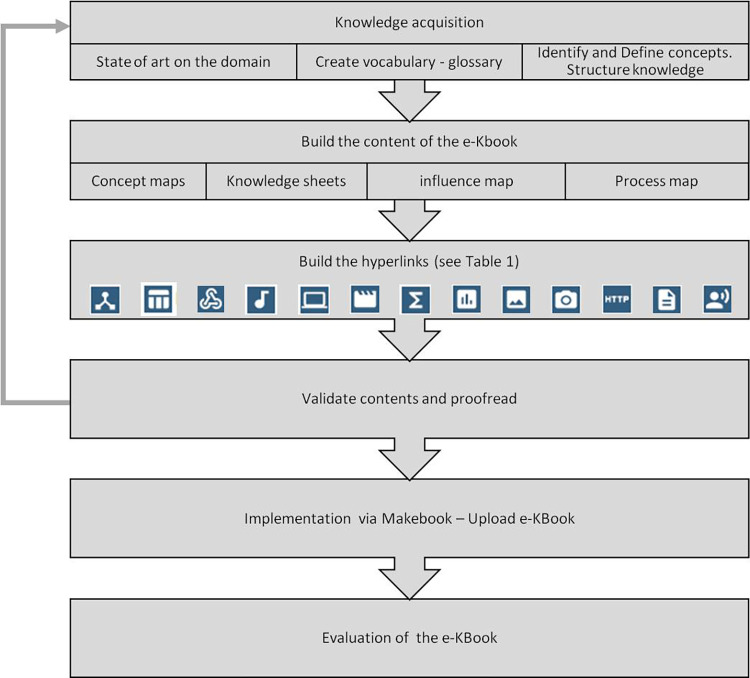
Design steps of an eK-Book.

Firstly, the end users of the eK-Book must be identified, as this allows the designer to calibrate the contents associated with the operational vocabulary and the way in which the content of the knowledge will be formalised and presented within the eK-Book. For instance, the use of overly scientific vocabulary could be detrimental to the appropriation of the eK-Book’s contents by future users. Identifying future users also makes it possible to assess the level of general knowledge on which to base the corpus of knowledge to be contained in the eK-Book. The definition of the upstream scope of the project makes it possible to decide to what level or depth of knowledge one wishes to go. This stage must be carried out in consultation with all the members of the project in order to reach a consensus approved by all the parties and to define a framework from which to build the methodology best suited to the project. Fig 7 displays the different steps of the eK-Book’s design.

The process of knowledge acquisition and expert knowledge elicitation [10, 41, 42] involves gathering the prior knowledge necessary for the understanding of a field, a study. It requires identifying existing platforms, tools, projects, ontology, thesaurus, compendium and experts related to the study domain. Taking into account the relevance of the knowledge to be transmitted in a transfer process is crucial. Indeed, the inclusion of non-useful and irrelevant knowledge leads to a reduced effectiveness of the knowledge transfer. The building of a common glossary is necessary when it is important for the vocabulary used in the knowledge book to be shared and understood by all: farmers, craftspeople, manufacturers, operators, scientists, etc. The knowledge gathered is then represented using either conceptual maps or a graph process or an influence graph or a knowledge sheet. With the help of the glossary/vocabulary, a list of concepts is proposed.

### Homepage design

**Fig 8:**
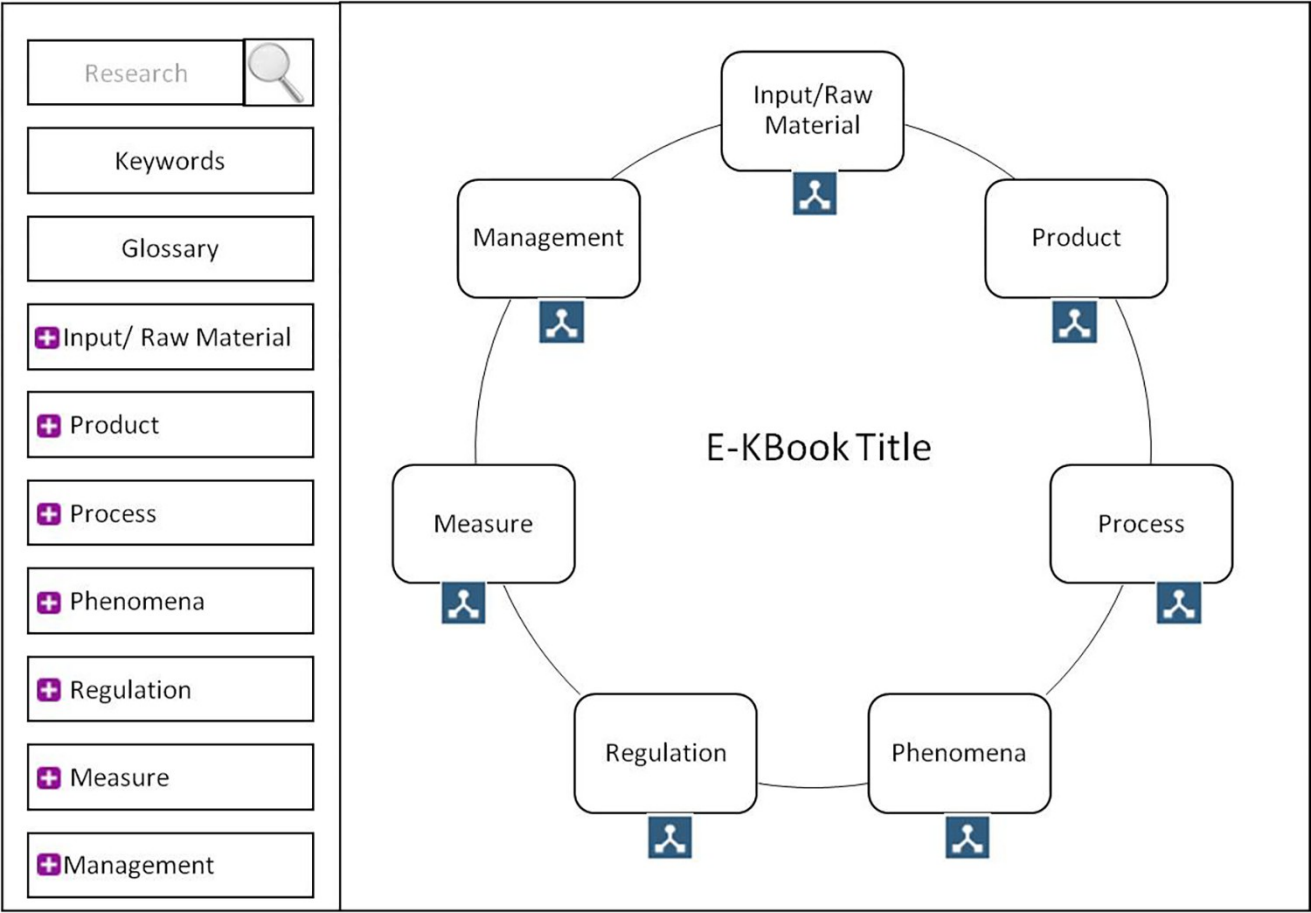
Section of a basic generic homepage.

The homepage is built to reflect the organisation of the main sections structuring the main inputs inside the eK-Book, i.e., the structure and visualisation of the table of contents for the first level of reading. With the help of the glossary/vocabulary, a list of main concepts is proposed. Fig 8 displays a basic section of a generic cover map.

### Cmaps design process

**Fig 9:**
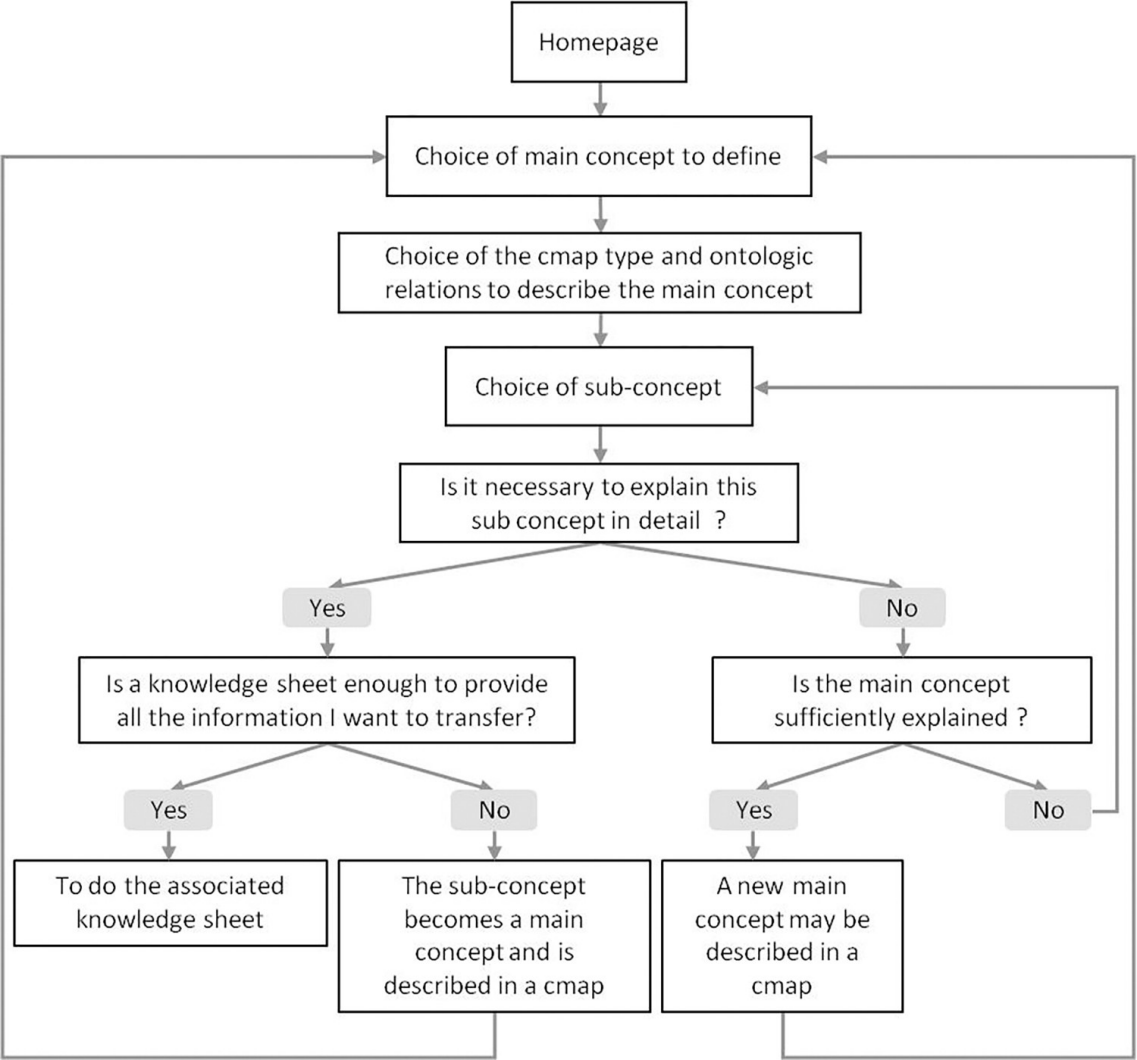
Flowchart of the cmap design process.

**Fig 10:**
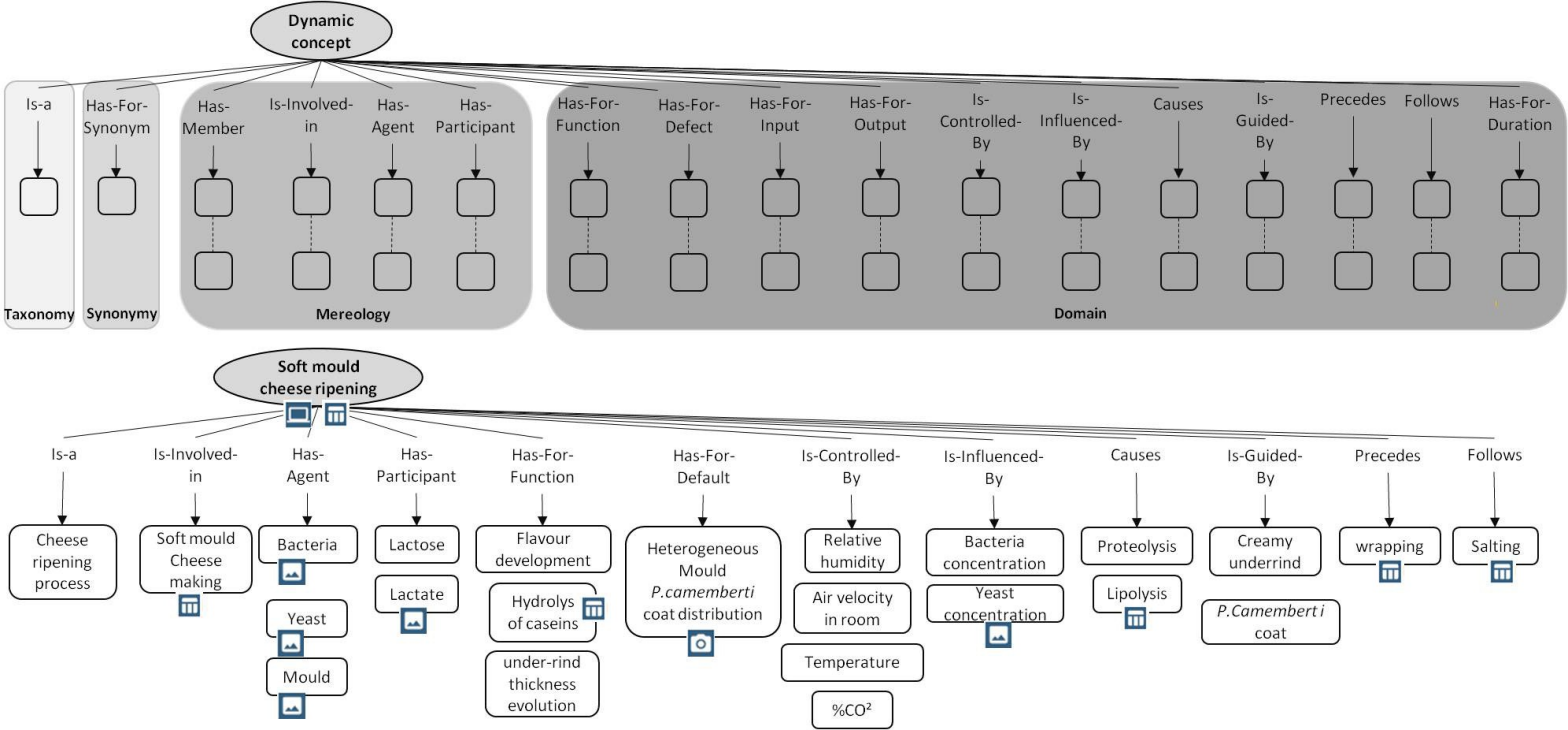
A generic canonical cmap to represent dynamic concepts (on the left) and a section of the instantiated cmap associated with the soft mould cheese ripening process (on the right).

**Fig 11:**
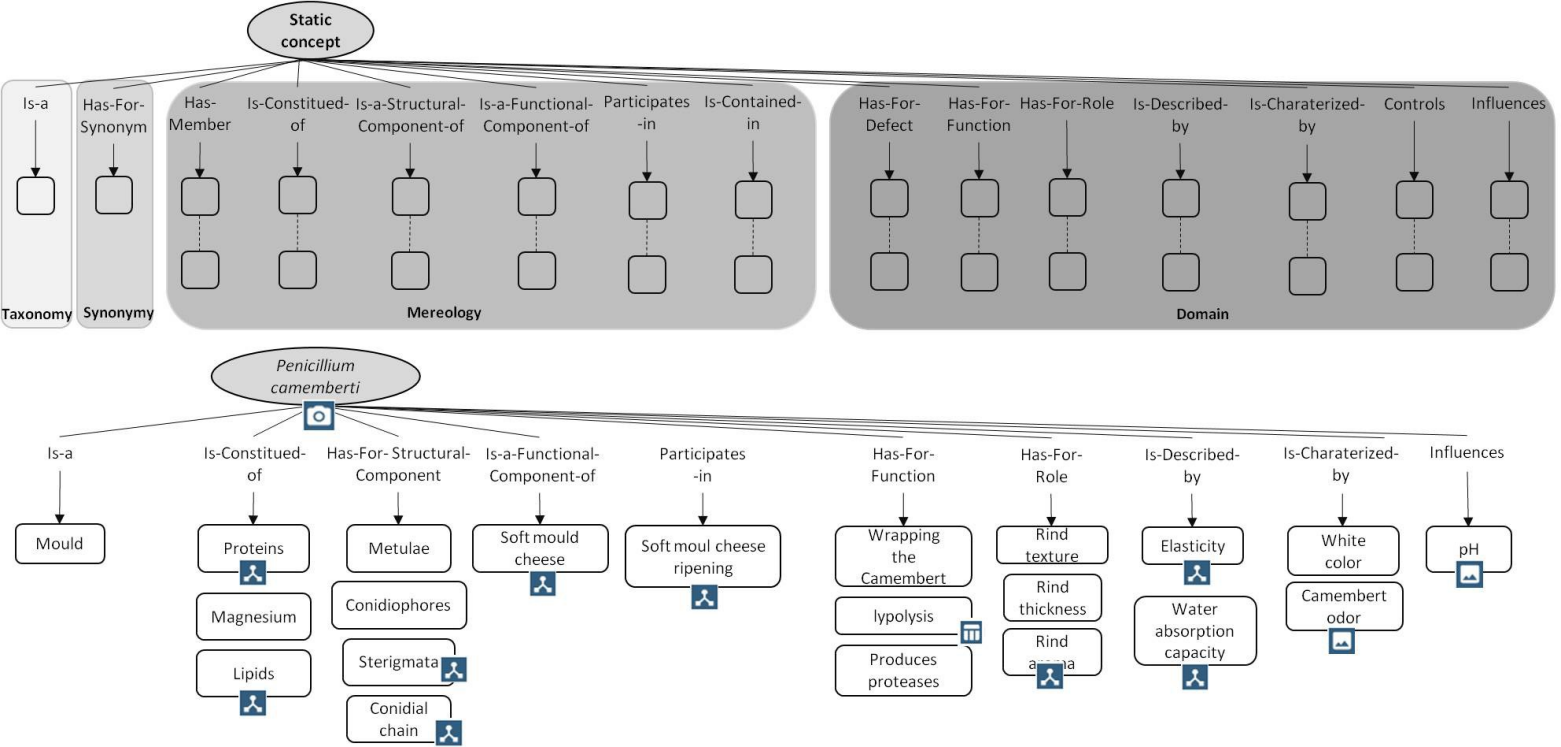
A generic canonical cmap to represent static concepts (on the left) and a section of the instantiated cmap associated with the mould *Penicillium Camemberti* (on the right).

Fig 9 displays the different steps to follow to build the set of cmaps. Each main concept identified is described by means of the canonical cmap displayed in Fig 2, using the relations listed in S1 and S2 Files. A Cmap has to be able to be read as a text without ambiguity. To help and guide the designer of the eK-Book, this work involves two types of canonical cmaps, one for static concepts such as materials, products, inputs, etc., and other for dynamic concepts such as phenomena or processes. Fig 10 displays the canonical conceptual map capable of representing all type of processes and an instantiation of this cmap explaining the soft mould cheese ripening process. Fig 11 displays a generic canonical cmap capable of representing all types of “static” concepts and a section of the instantiated cmap associated with the mould *Penicillium Camemberti*. This paper focuses on cmaps because they are the skeleton of the eK-Book, but similar processes may be implemented to design process maps, influence graphs and knowledge sheets.

### Implementation

**Fig 12:**
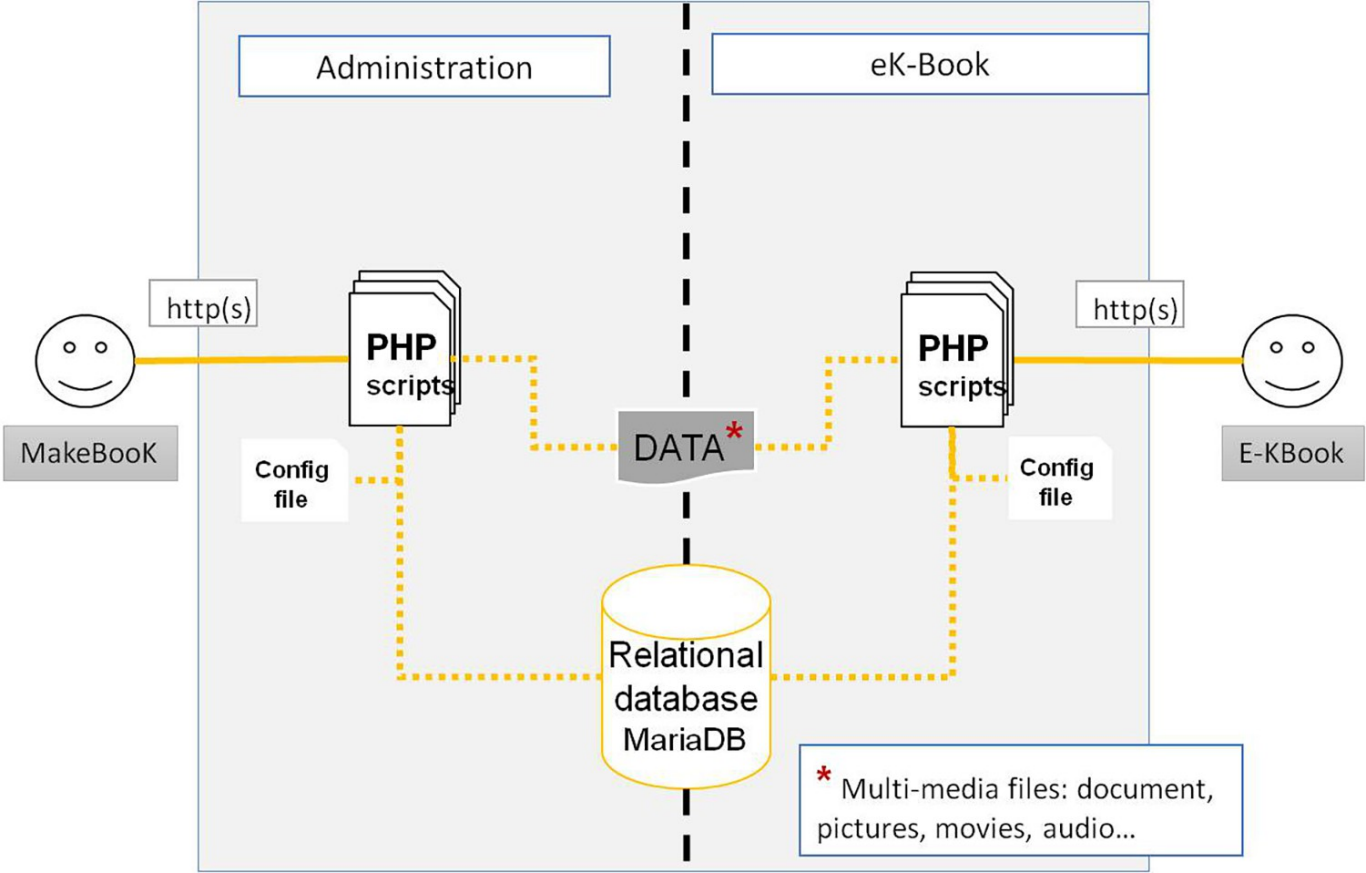
General architecture of the MakeBooK web application.

The eK-Book is implemented using the MakeBook© tool [43], which is an electronic knowledge book creation environment developed with Web 2.0 tools (HTML5, PHP 7.x MariaDB). MakeBook is an integrated solution designed to be easily usable by also those whose knowledge of computers is limited. Its web-oriented interface allows for use with modern browsers, making it multi-platform and multi-environment. It allows any users to directly create and visualize an ek-book; it is divided into two parts, as shown in Fig 12:

- An administrator part for the creation, editing and deletion of an eK-Book;
- A user part for browsing an eK-Book.

The administrator part provides a set of web forms able to store the whole eK-Book project’s data in a relational database. The user part can be likened to a website skeleton, which will be populated by the database and configured with an existing stored eK-Book project. This architecture allows the logical and physical decoupling of all software parts to minimise, at minimum, the following concerns:

- using web clients;
- taking into account the heterogeneity of servers;
- breaking the strong coupling between data and algorithms.

#### Administration part

Creating and editing (or deleting) an eK-Book project with MakeBooK© is a collective (not collaborative) process between administrators, but it also requires authentication. Three levels of administrator can be granted:

- Project Administrator: administrator with all rights to add, edit and delete documents and their links (C-maps, knowledge sheets, links, references, keywords, authors, etc.) in each eK-Book, including the complete management of the eK-Book’s projects, the ability to create administrators and assign their rights to access projects and the ek-book’s menu management;
- Book Administrator: administrator with all rights except ek-book project management and administrator management;
- Documents Administrator: administrator with only the rights to add, edit and delete documents and their peripheral data (e.g., Author, Partner).
- An administrator can also create an authenticated user without any administration level, called a “Reader” (LEC): this user is only allowed to browse the eK-Book and is subject to content restrictions: a readable document and a user must be members of the same group.

**Fig 13:**
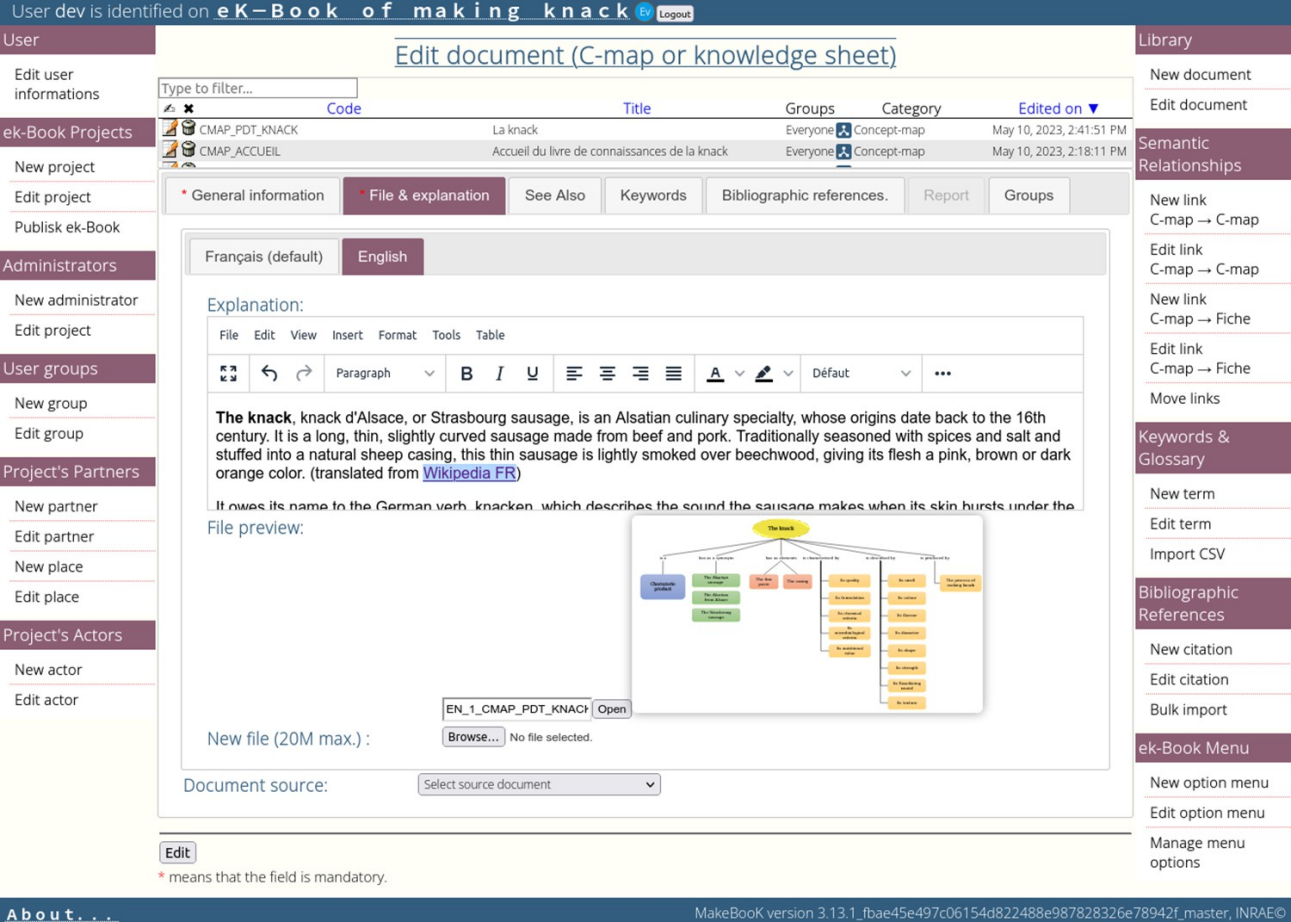
Example of an enhanced web form to facilitate the user experience.

Pragmatically, the process of building an eK-Book needs to:

1. design and manage files (representing concept maps, process maps, influence graphs or hypermedia knowledge sheets) generated by common software in standard file formats (images, documents, videos, sounds, etc.);
2. associate relevant data with them, as shown in Fig 4;
3. create navigational hyperlinks between them through dedicated and user-friendly web forms (see Fig 13).

To address these features, a simplified electronic document management system has been developed. An eK-Book’s document stores a file and the administrator decides what kind of eK-Book object it will represent (see Fig 1) according to its file format. For example, the interface will propose that a jpeg image can be either a concept-map or a knowledge sheet (“Illustration”, “Schematics”, “Photography”, etc.), but mpeg4 video content can only be associated with a "Movie" type of knowledge sheet. Next, the administrator can tag the document with authors (AKA Actors), keywords, citations and “See Also” links. These elements appear in selection lists and have their own information, e.g., an Actor must have a first and last name, but if desired, a contact address, affiliated structures, etc. may be given. Keywords can be entered with a definition to automatically build a glossary in an eK-Book. All data are manageable with dedicated web forms to avoid duplication and encourage the reuse of linked data. Some improved web forms are available to easily build the hypertext network of the eK-Book. There are web forms to:

- visually place, move, change, delete navigational hyperlinks on Cmap;
- directly move all hyperlinks on a Cmap;
- create menu entries allowing direct access to a Cmap or knowledge sheet and arrange them as a hierarchical menu displayed on the left of the eK-Book.

Finally, administrators can provide additional information on the eK-Book project, visible to users, such as a textual description, an “About” page, or a logo. They can also define the eK-Book interface’s default language as documents, project, menus and keywords are localisable: a tabbed interface (per language) is implemented on every translatable field of its web forms.

#### User part

**Fig 14:**
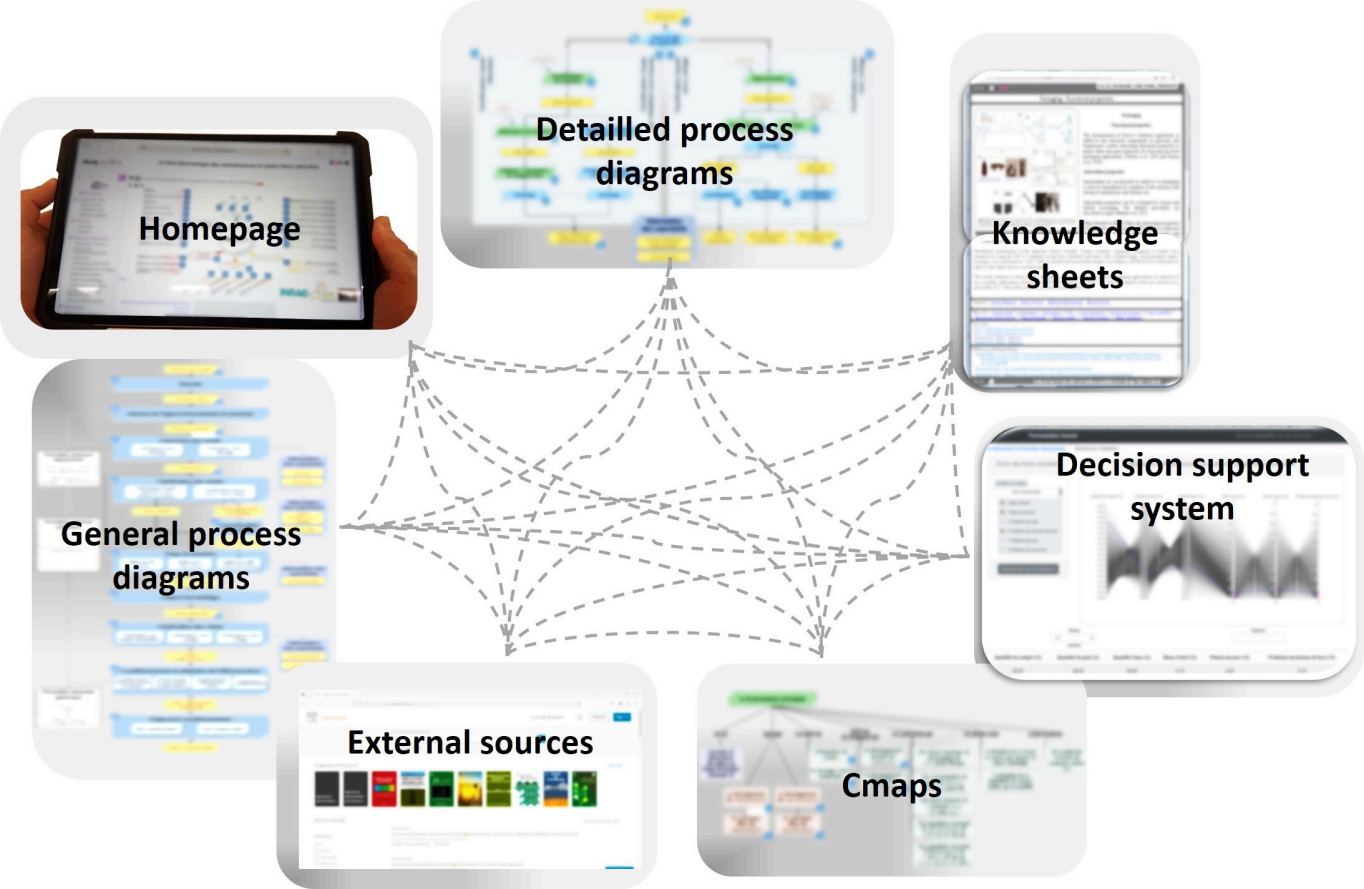
Overview of the different documents in an implemented eK-Book.

The results of the implementation with MakeBook can be viewed directly by browsing the eK-Book via a modern browser and can be accessed through smartphones and tablets. Users will not be forced to read knowledge in a sequential linear way. The interface affords them the ability to navigate (browse) freely in the hypertext and choose between different pathways in the content. This freedom is relevant for learners to access specific information or to construct their own reading path according to their interests or needs. From a cognitive point of view, the hypertext structure is very similar to the human cognitive architecture and allows concepts to be described within different contexts. This feature improves cognitive flexibility and increases the transfer of learned concepts. Fig 14 displays an overview about the different documents present in an eK-Book and the interacting network between all the components of the eK-Book. The only restriction is that, by default, the identified users can only browse documents which belong to the same groups as them, enabling information to be partitioned between users of different groups.

**Fig 15:**
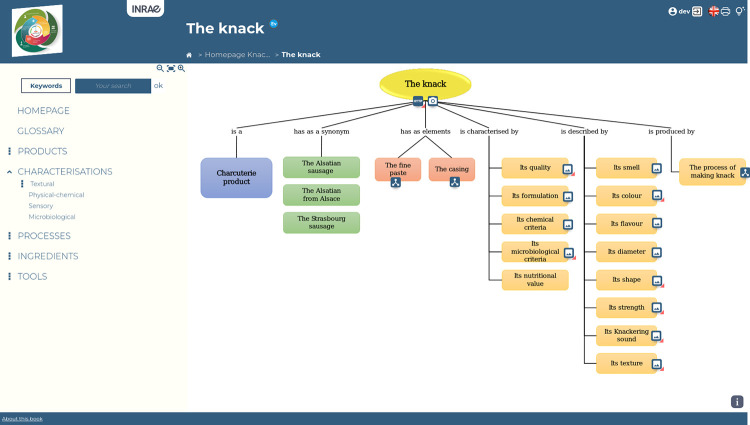
General interface of an eK-Book.

The interface of the eK-Book is divided into three visible sections: a header, a left section and a middle section (Fig 15).

**Fig 16:**
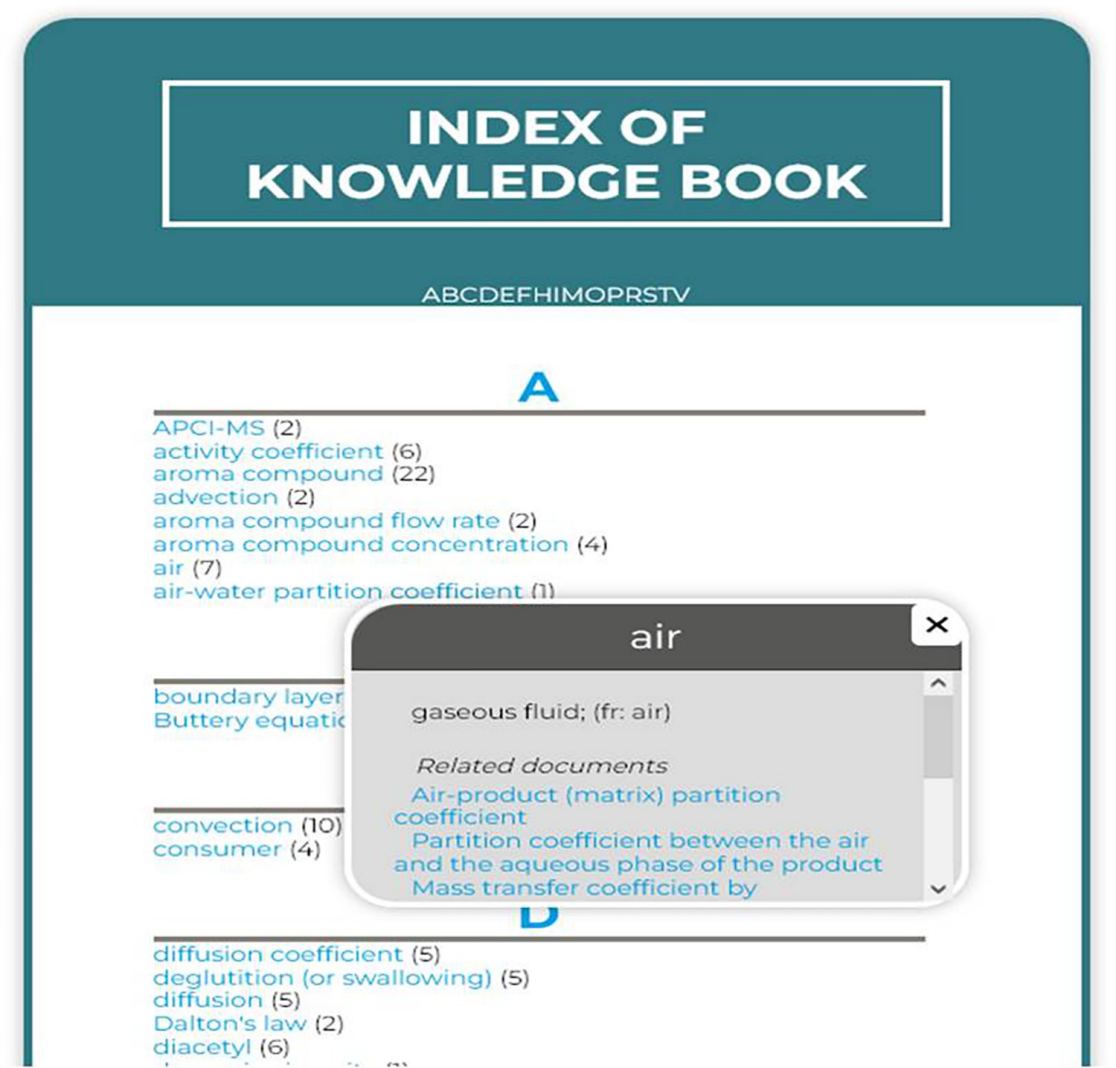
Glossary index.

The header displays the classical features of a website: the logo of the eK-Book’s project (a hyperlink to the homepage), the main title of the Cmap currently displayed, a breadcrumb trail, a user information zone with a reminder of the user’s groups, a disconnect button and a dynamic language changer. Two shortcuts are available: a brief interactive tutorial to explain how to use the eK-Book and a print button. The left section is composed of two parts. The top part has a button to display a Tag Cloud of keywords and a search bar. The Tag Cloud is dynamically generated and the keywords are displayed in sizes that are more visible the more they are associated with documents. Clicking on a keyword triggers a search on the keyword in the search bar. The search bar does a search both by keywords and textual occurrences. It leads to a result page presenting a list of Cmaps or knowledge sheets, etc., associated with the exact corresponding keyword given, and another list where the subject of the search appears in textual fields of document such as title, explanation or citation (see Fig 3). The rest of the left section is occupied by the hierarchical menu configured by MakeBook’s administrator. It is a collapsing menu to directly point to a Cmap, process map or influence graph and, in a window, displays the eK-Book’s glossary index (Fig 16).

Finally, the middle section displays Cmaps, process maps or influence graphs with their navigational hyperlinks. By clicking on a knowledge sheet’s pictogram, a pop-up window is displayed above the middle section to read its content; otherwise, it opens a new browser tab for external resources or simulation models. In its bottom-right corner, an information button flips the middle section to display some metadata of the current document (title, dates, groups of the document, textual description, references) and lists incoming and outgoing navigational hyperlinks.

## Evaluation of the eK-Book’s ability to transfer knowledge

**Fig 17:**
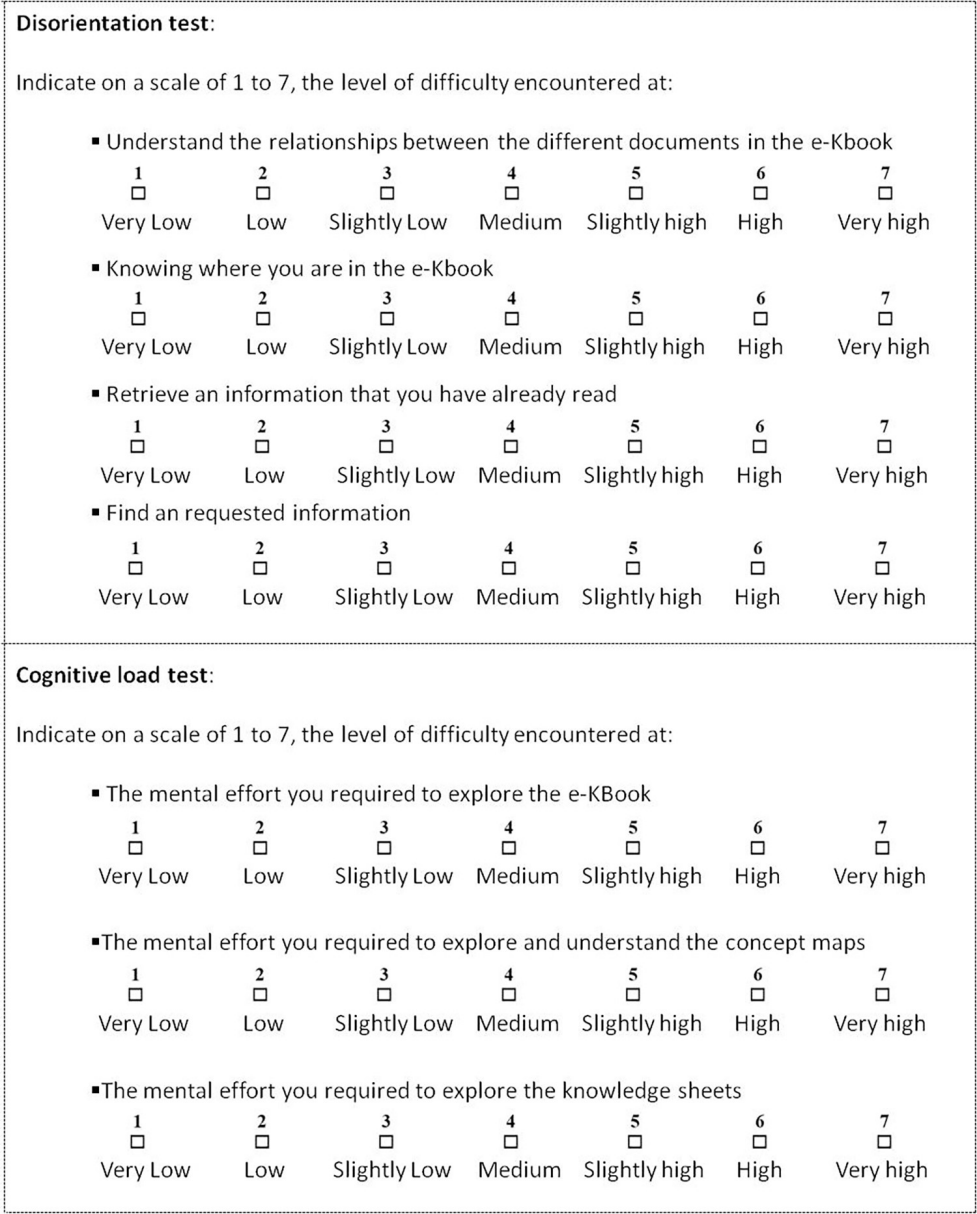
Section of questions from the disorientation and cognitive load tests.

Knowledge transfer is defined as the process by which knowledge is “transmitted” from a source to a target (e.g., future users, learners); it involves the transmission and absorption of knowledge [44, 45], meaning that ‘*transfer*’ = ‘*transmission*’ + ‘*absorption*’. Its effectiveness is closely linked to the degree to which the recipient understands the transferred knowledge, as well as the cognitive load required and the disorientation that the transmission channel may generate. The capacity of the eK-Book to transfer knowledge is evaluated by means of an aggregation of three measures, namely cognitive load, disorientation and understanding, through single select multiple-choice questions. The lower the cognitive load and disorientation, the easier the transfer capacity. The higher the degree of comprehension, the more effective the transfer capacity. The degree of understanding is determined through two tests, based on single select multiple-choice questions, conducted before (pre-test) and after (post-test) navigation in the eK-Book [46]. Cognitive load and disorientation are measured using the self-rating technique based on a 7-point mental effort rating scale [47] ranging from 1 (very low) to 7 (very high). A mental effort rating scale is easy to use (non-intrusive) and provides a good indication of cognitive load and disorientation. The tests then consist in assigning a rating to states, as shown in Fig 17.

The test protocol to evaluate the transfer capacity of an eK-Book comprises the following steps:

- Step 1: General presentation of the eK-Book and instructions,
- Step 2: Complete the information sheet indicating the level of qualification and skills in the field,
- Step 3: Pre-test single select multiple-choice questions n°1,
- Step 4: Free navigation using the eK-Book for 20 minutes,
- Step 5: Cognitive load and disorientation tests,
- Step 6: Scripted navigation for 20 minutes. Users have to find requested information by using the eK-Book. The aim is to evaluate the usability and efficiency of the eK-Book and thus to ensure that the structure of the eK-Book allows users to find and compile information.
- Step 7: Post-test single select multiple-choice questions n°2,
- Step 8: Cognitive load and disorientation tests.

Led and supervised by a knowledge engineer in charge of guiding debates and ensuring the cohesion and homogenisation of the contents built, two eK-Books have been designed and tested in the cheese sector and the butchery and cold meat sector. The URLs of each eK-Book associated with the online questionnaire (see example in Fig 17) and the test protocol were sent by email to different stakeholders. Each participant was free to carry out the test whenever they wished. The analysis of the replies to the questionnaire regarding the evaluation of the Cheese ek-book and the butchery and cold meat eK-Book were analyzed anonymously.

### Evaluation of the eK-Book on butchery and cold meat products

An eK-Book was implemented on the manufacture of butchery and cold meat products, and more precisely the manufacture of Knack sausages and meat cutting. This eK-Book contains 40 cmaps and 149 knowledge sheets, of which 21 contain short subtitled videos. This book also features two simulation tools for:

1. estimating the texture of a Knack according to the type of protein and the type of gut used, or estimating the type of gut to use according to the expected texture,
2. formulating a Knack recipe according to the types of meat and binders used.

The use of the book was tested with different stakeholders from technical centres, industrial firms producing meat-based food products and a world-leading manufacturer of industrial ingredients. Twenty-two people belonging to three different sectors took the test to evaluate the capacity of the eK-Book to transfer knowledge and know-how.

**Fig 18:**
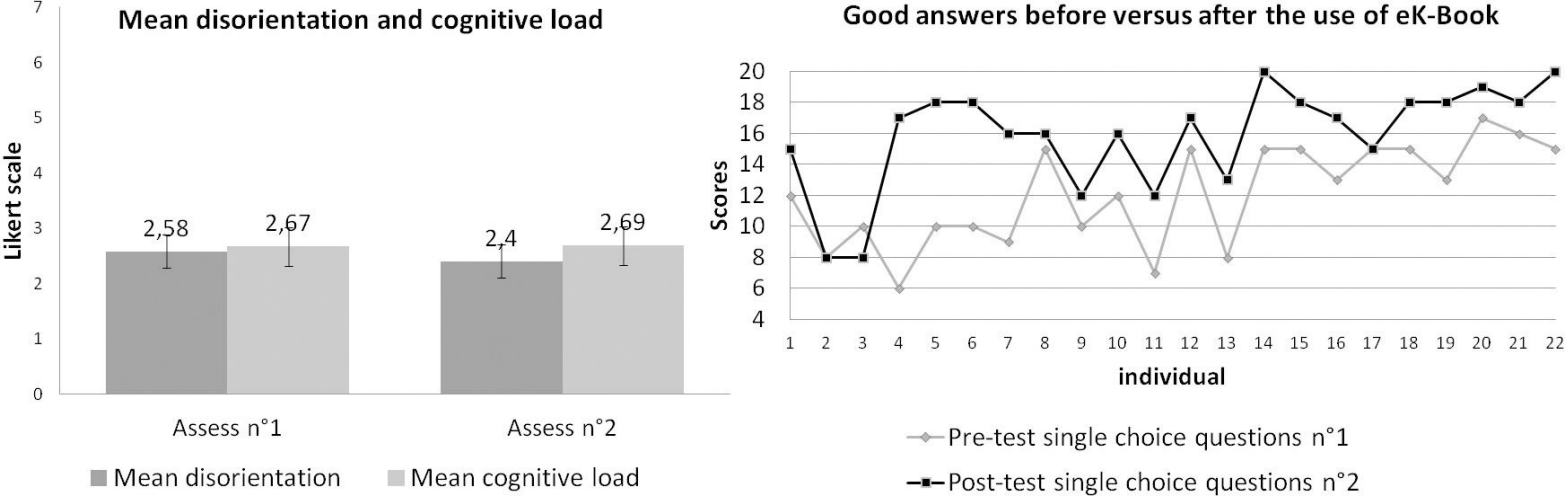
(On the left) mean disorientation and mean cognitive load at step 5 (Assess n°1) and step 8 (Assess n°2). (On the right) number of right answers from the twenty-two people before versus after navigation in the eK-Book.

Fig 18 displays (on the left) the evolution of the mean disorientation and the mean cognitive load of the users participating in the test. On the right, Fig 18 displays the number of right answers from the twenty-two people before versus after navigation in the eK-Book. The results show very positive feedback about the training tool, knowledge acquisition and disorientation and cognitive load of users. Except for individuals 2, 3, and 17, all users improved their level of knowledge; for instance, individuals 4 to 7 considerably increased their scores. The mean disorientation and the mean cognitive load are relatively low and stable irrespective of the spent time on the eK-Book website.

### Evaluation of the eK-Books on PDO and PGI cheeses

An eK-Book was implemented on the manufacture of PDO and PGI cheeses, containing 801 knowledge sheets, 293 cmaps and 8 process graphs. The eK-Book was tested with operators, students and trainers from three different chains. Fifteen people belonging to three different chains took this test.

**Fig 19:**
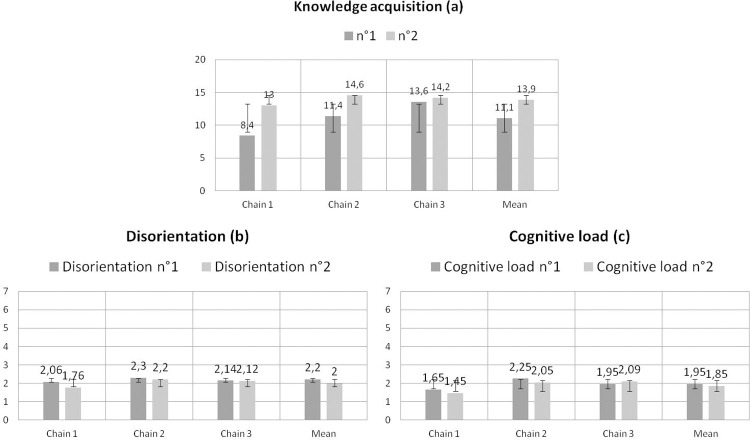
Test results of knowledge acquisition, disorientation and cognitive load conducted among 15 people in the cheese sector: y-axis (a) (*resp*. (b) and (c)) represents the number of right answers (mean value) (*resp*. mental effort) in the three chains and on average.

Fig 19 displays the test results: (a) the multiple-choice questions to assess knowledge acquisition, and the self-evaluation of (b) disorientation and (c) cognitive load, conducted among 15 people in the cheese sector. The average knowledge acquisition score was 11.1/20 before using the eK-Book and 13.9/20 after, i.e., a gain of 2.8 points. This result suggests that during navigation, participants were able to identify, understand and acquire new knowledge about the study area from the knowledge book. The average scores for disorientation and cognitive load were respectively 2.2 and 1.95 after the free navigation. These scores improved after the scripted navigation, decreasing to 2 for disorientation and 1.85 for cognitive load. These results suggest that during the navigation, the participants managed to navigate the content without a significant cognitive load, which ranged from very low to low; nor disorientation, which can be considered as negligible with an average score ranging from low to slightly low.

## Conclusion

Given the need to provide knowledge transfer tools in a context of numerical and digital transition, eK-Books are efficient tools to collect, structure and remobilise knowledge and know-how. They are designed for expert or novice operators (technical departments in a sector or a company), manufacturers, sector coordinators or plant managers, as well as trainers and learners (initial and continuing education). Indeed, the web navigation from cmap to cmap provides more in-depth explanations of specific concepts or the exploration of new concepts. EK-Books may be easily updated and upgraded without any negative effects on their overall structure and may have access restrictions with identification in order to control the compartmentalisation of information. EK-Books allow users and designers to preserve knowledge and skills in various sectors. The methodology implemented using MakeBook© allows any user who wishes to save their knowledge to be autonomous and to minimise the intervention of knowledge engineers. The creation of an eK-book may be modulated and adapted to different objectives at different scales (short term vs. long term, training vs. decision support etc.). This paper highlights the usability and effectiveness of eK-Books in transferring knowledge in the agri-food field. Regardless of the eK-Book, tests have shown that disorientation and cognitive loads remain low. The results of the evaluation show that the navigation is quite intuitive and that knowledge acquisition is significant for more than 95% of users. The cheese eK-Book is currently being used and enriched in 2023 within 13 cheese regions of France (Comté, Morbier, Mont d’Or, Bleu de Gex, Gruyère IGP, Beaufort, Abondance, Reblochon, Emmental, Cantal, Salers, Bleu de Vercors and Camembert de Normandie). This enrichment has led to the creation of a French association complying with the French 1901 law (www.docamex.fr) where a knowledge engineer is responsible for deploying this digital platform to many cheese sectors and dairy industries. He is in charge of supporting and training referents who wish to create their own book. He extends the approach upstream in the chain (milk production, breeding, etc.) where these issues of capitalization and transmission of knowledge and know-how are also significant. The genericity of MakeBook©, the eK-Book’s engine, allows it to be deployed in any sector where knowledge has to be saved and transferred. Learning is a lifelong process, the eK-Books provide an adapted environment in which interactive and personalized continuous learning programs could be implemented by industrial firms that cater to individuals at different stages of their careers or lives. They offer a channel for informal learning more and more encouraged by organizations where the learners are in total control and set their own goals and objectives. EK-Books solution also ensures equitable and fair access to information breaking down barriers to education and opportunities, promoting social equity. They improve access to education and skill development, empowering individuals from diverse backgrounds. The tool encourages collaborations between diverse fields and industries facilitating the exchange of knowledge and expertise being able to lead to innovative solutions. The versatility of eK-Books plays a crucial role in sharing, updating and upgrading knowledge. Their architecture facilitates as well as the integration and the dissemination of technologies as practices as policies, contributing to a more sustainable future. However, irrespective of the existing tools, it is important to be aware that the process of knowledge elicitation and representation remains time-consuming.

## Supporting information

S1 Checklist Human participants research checklist. (DOCX)

S1 File Mereological relations. (DOCX)

S2 File Domain relations [39, 48–55]. (DOCX)
