# Supplementary material for: Electronic knowledge books (eK-Books) as a medium to capitalise on and transfer scientific, engineering, operational, technological and craft knowledge
Source: PLoS One. 2024 May 17;19(5):e0299150. doi: 10.1371/journal.pone.0299150 (PMC11101106; doi:10.1371/journal.pone.0299150)
Supplement: S1 Checklist — (DOCX) [file pone.0299150.s001.docx]

**Human Participants Research Checklist**

***Complete the following if your study involved human participants or human participants’ data. These questions should be addressed for prospective and retrospective studies.***

1. Did you obtain ethics approval for this study?
   - If yes, please upload (file type “Other”) the original approval document you received from your ethics committee. If the original document is in another language, please also provide an English translation.

___ Uploaded ___ N/A

- - If you did not obtain ethical approval, please explain why this was not required below.

Two eK-Books tested online in the cheese sector and the butchery and cold meat sector. The urls of each eK-Book associated with the online questionnaire (see exemple in Fig. 16) and the test protocol were sent in 2022 by email to different stakeholders (15 for the cheese eK-Book and22 for the butchery and cold meat eK-Book) . Each participant was free to carry out the test whenever they wished. The analysis of the replies to the questionnaire was done such that individual subjects cannot be identified. That means that the replies to the questionnaire regarding the evaluation of the Cheese ek-book and the butchery and cold meat eK-Book were analyzed anonymously. For these reasons, ethical approval was not required. Moreover, this part does not correspond to the main contribution of our article. This is now specified in the section “Evaluation of the eK-Book’s ability to transfer knowledge”

1. If you prospectively recruited human participants for the study – for example, you conducted a clinical trial, distributed questionnaires, or obtained tissues, data or samples for the purposes of this study, please report in the Methods:
   1. the day, month and year of the **start and end** of the recruitment period for this study.
   2. whether participants provided informed consent, and if so, what type was obtained (for instance, written or verbal, and if verbal, how it was documented and witnessed). If your study included minors, state whether you obtained consent from parents or guardians. If the need for consent was waived by the ethics committee, please include this information.

___ Completed _x_ N/A

1. If you are reporting a retrospective study of medical records or archived samples, please report in the Methods section:
2. the day, month and year when the data were accessed for research purposes
3. whether authors had access to information that could identify individual participants during or after data collection

___ Completed _x_ N/A
