# Supplementary material for: Electronic knowledge books (eK-Books) as a medium to capitalise on and transfer scientific, engineering, operational, technological and craft knowledge
Source: PLoS One. 2024 May 17;19(5):e0299150. doi: 10.1371/journal.pone.0299150 (PMC11101106; doi:10.1371/journal.pone.0299150)
Supplement: S1 File — (DOCX) [file pone.0299150.s002.docx]

**Annexe I : Mereological relations**

| **Relation** | **Definition** | **Inverse** | **Example** |
| --- | --- | --- | --- |
| *C* ***Is-Member-of*** C’ where C’ is a social object and C is a social or a physicalobject. | Relates an aggregation of physical objects (or non physical objects) into a whole existentially dependent on its parts | *Has-member* | Factory ***Has-Member***- employees |
| C ***Is-Constituted-of*** C’ where C’ is a material and C the physical object | Physical object related to an amount of matter it is made of | *Constitution of* | Cheese **Is-*Constituted-of*** milk fat |
| C ***Is-a-Structural-Component-of*** C’ where C and C’ are physical or not physical objects | Relates structural components of the whole | *Has-For-Structural- Component* | Shelf ***Is-a-S- Component-of*** cupboard |
| C  **Is-a*-Functional-Component-of***C’ where C and C’ are physical or not physical objects | Relates structural component C of the whole C’ where C’ becomes dysfunctional or does not exist if the function of C cannot be executed | *Has-For-Functional-Component* | Heart ***Is-a-F-Component*-of** body |
| *C* ***Participates-in*** *P* where C is an entity and P a phenomenon | Relates an entity C to the phenomenon P  it participates in | *Has- Participant* | Protein ***Participates in*** *milk* coagulation |
| *P* ***Has-Agent*** *C* where C is an entity and P a phenomenon or a process | Relates an entity C to the phenomenon or process P  it participates in and is causally responsible for P | *Is-Agent* | Proteolysis ***Has-Agent*** enzyme |
| P ***Is-involved-in*** P’ where P and P’ are processes | Link between process step and the process | *Involves* | Cheese ripening ***Is- involved-in*** cheese making |
| C ***Is*_*Located_in*** C’  C and C’ s are regions | Relates two independent spatial regions in which every C at any given time occupies a spatial region which is part of the region occupied by some C’ at the same time | *I*s-*Location-of* | my brain ***Is Located-in*** my head |
| C ***Is-Contained-in*** C’ where C and C’ s are regions | Every C at any given time is located in C’ but shares no parts in common with some C’ at the same time | *Contains* | Antibiotic ***Is-Contained-in*** milk |
| C ***Is-A-Sub-Quantity-of*** C’ where C and C’ are materials | Relates a smaller part-amount of matter C to a whole-matter C’ where the amounts of matter are either of the same nature or the part is a different type of matter than the whole, | *Is-A-Power-Quantity-of* | A glass of water ***Is-A-Sub-Quantity-of*** bottle of water |
