# Supplementary material for: Electronic knowledge books (eK-Books) as a medium to capitalise on and transfer scientific, engineering, operational, technological and craft knowledge
Source: PLoS One. 2024 May 17;19(5):e0299150. doi: 10.1371/journal.pone.0299150 (PMC11101106; doi:10.1371/journal.pone.0299150)
Supplement: S2 File — (DOCX) [file pone.0299150.s003.docx]

**Annexe II: Domain relations**

| **Topic** | **Relation** | **Definition** | **Inverse** | **Example** | **Reference** |
| --- | --- | --- | --- | --- | --- |
| Measure - Observation | S ***Made-Observation*** O where S is a sensor and O an observation | Relation between a Sensor and an Observation made by the Sensor | ***Made-by-Observation*** | pH-meter **Made**-***Observation*** 8.2 | [48] |
|  | S ***Observes*** O where S is a sensor and O an observable quality (property, characteristic) | Relation between an observable property and the sensor able to observe it | ***Is-Observed-by*** | pH-meter ***Observes*** pH |  |
| Description | C ***Has-For-Function*** F where C is an entity and F a function | A relation between an entity C and the function F played by C in some process or phenomenon that describes rights and obligations of C in some particular circumstance | ***Is-Function-Of*** | Amylase ***Has-For-Function*** to break starch into sugar | [49] |
|  | C ***Has-For-Role*** R where C is an entity and R a role | A relation between an entity C and the role R played by C in some process or phenomenon | ***Is-Role-of*** | Amylase ***Has-Role*** starch hydrolysis |  |
|  | C ***Has-For-Quality*** Q where C is an entity and Q a quality | Relates any aspect of an Entity (but not a part of it), which have to be respected and cannot exist without that Entity. | ***Is-Quality-of*** | the bread crumb ***Has-For-Quality*** a slightly elastic texture | [50] |
|  | C ***Is-Described-by*** D where C may be a physical or not physical object and D a description | Relates any descriptor, property of an entity which may change with external factors | ***Describes*** | Cider ***Is-Described-by*** its colour |  |
|  | C ***Is-Characterized-by*** D where C may be a physical or not physical object and D a characteristic | Relates any characteristic, property of an entity which is unique to it and independent of all external factors | ***Characterizes*** | Milk ***Is-Characterized-by*** its white colour |  |
| Process/Phenomenon control | P ***Has-For-Input*** C where P is a process and C is a material | p has input c iff: p is a process, c is a material entity, c is a participant in p, c is present at the start of p, and the state of c is modified during p. | ***Is-Input-of*** | Concrete mixing ***Has-For-Input*** Cement | [39] |
|  | P ***Has-For-Output*** C where P is a process and C is a material | p has output c iff c is a participant in p, c is present at the end of p, and c is not present in the same state at the beginning of p. | ***Is-Output-of*** | Concrete mixing ***Has-For-Output*** Concrete |  |
|  | C ***Controls*** P where P is a process and C is a control, agent | Relates a control, agent C to a process or phenomenon P that may alter the flow, behaviour of p | ***Is-Controlled-by*** | Temperature ***Controls*** the fermentation | [51] |
|  | C ***Influences*** C’ where C may be entity, activity, or agent … | Relates an entity, activity, or agent C to another one C’ which has the capacity to have an effect on the character, development, or behaviour of C’ | ***Is-Influenced-by*** | Milk composition ***Influences*** milk quality | [52] |
|  | P ***Is-Guided-by*** T where P is a process or phenomenon and T a targer | Relates a target to be achieved by a process | ***Guides*** | Kneading ***Is-Guided-by*** the desired state of the dough |  |
| Temporality | P ***Precedes*** P’ where P and P’ are process or phenomenon | x precedes y if and only if the time point at which x ends is before or equivalent to the time point at which y starts. Formally: x precedes y iff ω(x) <= α(y), where α is a function that maps a process to a start point, and ω is a function that maps a process to an end point. | ***Follows*** | Ingestion ***Precedes*** digestion | [39] |
|  | C ***Derives_from*** C’ where C and C’ are material object | a relation between two distinct material entities, the new entity and the old entity, in which the new entity begins to exist when the old entity ceases to exist, and the new entity inherits a significant portion of the matter of the old entity | ***Derives_into*** | Fatty acide ***Derives_from*** triglyceride |  |
|  | C ***Has-For-Duration*** D where C is a temporal entity and C is duration | Relates a temporal entity, event or activity, or thing to a duration expressed as a scaled value | ***Is-Duration-of*** | Soft mould cheese ripening process ***Has-For-Duration*** 15 days | [53] |
| causality | P ***Causes*** P’ where P and P’ may be phenomenon or process | If P (cause) occurs, then P’ (effect) occurs | ***Is-Cause-by*** | crushing ***Causes*** The reduction of particle size | [54] |
|  | C ***Influences*** P’ where C is an entity and P’ is a phenomenon or process | Relates an entity that may positively influences or negatively influence a phenomenon or a process P’ | ***Is-Influenced-by*** | The presence of inhibitors ***Influences*** the velocity of fermentation | [54] |
| Planning/Action | C ***Has-Corrective-Action*** A where C is a defect and A an action | Relates a long-term action A to correct a defect | ***Is-Corrective-Action*** | Presence of damaging mould during ripening ***Has-Corrective-Action*** check cleaning ripening room | [55] |
|  | C ***Has-Compensatory-Action*** A where C is a defect and A an action | Relates an immediate action A to at least reduce the defect C | ***Is-Compensatory-Action*** | Presence of damaging mould during ripening ***Has- Compensator-Action*** remove contaminated cheeses of ripening room |  |
|  | C ***Has-Recommendation-Action*** A where C is a quality entity and A an action | Relates an action A to be carried out to maintain the particular quality standard of the product C | ***Is-Compensatory-Action*** | Presence of damaging mould during ripening ***Has- Compensatory -Action*** remove contaminated cheeses of ripening room |  |
